# Recombinant Influenza Vaccines: Saviors to Overcome Immunodominance

**DOI:** 10.3389/fimmu.2019.02997

**Published:** 2020-01-10

**Authors:** Nimitha R. Mathew, Davide Angeletti

**Affiliations:** Department of Microbiology and Immunology, Institute of Biomedicine, University of Gothenburg, Gothenburg, Sweden

**Keywords:** influenza A virus, immunodominance, vaccines, B cells, antibodies

## Abstract

It has been almost a decade since the 2009 influenza A virus pandemic hit the globe causing significant morbidity and mortality. Nonetheless, annual influenza vaccination, which elicits antibodies mainly against the head region of influenza hemagglutinin (HA), remains as the mainstay to combat and reduce symptoms of influenza infection. Influenza HA is highly antigenically variable, thus limiting vaccine efficacy. In addition, the variable HA head occupies the upper strata of the immunodominance hierarchy, thereby clouding the antibody response toward subdominant epitopes, which are usually conserved across different influenza strains. Isolation of monoclonal antibodies from individuals recognizing such epitopes has facilitated the development of recombinant vaccines that focus the adaptive immune response toward conserved, protective targets. Here, we review some significant leaps in recombinant vaccine development, which could possibly help to overcome B cell and antibody immunodominance and provide heterosubtypic immunity to influenza A virus.

## Introduction

Influenza viruses belong to the family of Orthomyxoviridae and consists of A, B, C, and D types. Types A and B are currently circulating among humans (1–4). Influenza causes significant morbidity (30–50 million cases yearly) and mortality, with infection-associated respiratory deaths in the range of 4–8.8 per 100,000 individuals, posing heavy socioeconomic burden to society (5). Annual vaccination remains as the mainstay to prevent influenza infection, but, according to Centers for Disease Control and Prevention, it is effective only in 20–70% of the population, depending on season (6). Based on antigenic and phylogenetic properties of influenza surface glycoproteins, hemagglutinin (HA), and neuraminidase (NA), there are 18 HA (H1–H18), and 11 NA (N1–N11) Influenza A virus (IAV) serotypes and two influenza B of B/Victoria and B/Yamagata lineages (7, 8). HA is further divided into two phylogenetic groups. The current seasonal flu vaccines are either trivalent or quadrivalent containing HA from circulating H1N1, H3N2, and B/Victoria lineage or both influenza B lineages (9). IAV possess an error prone RNA polymerase, which results in mutations in surface antigens, leading to antigenic drift and antibodies being no longer effective. Therefore, it is necessary to update and administer vaccines every year by forecasting the drifted strains. In addition, the annual vaccination becomes ineffective during pandemic outbreaks, in which a new viral strain of zoonotic origin acquires the ability to replicate in humans (10, 11).

**Figure 1:**
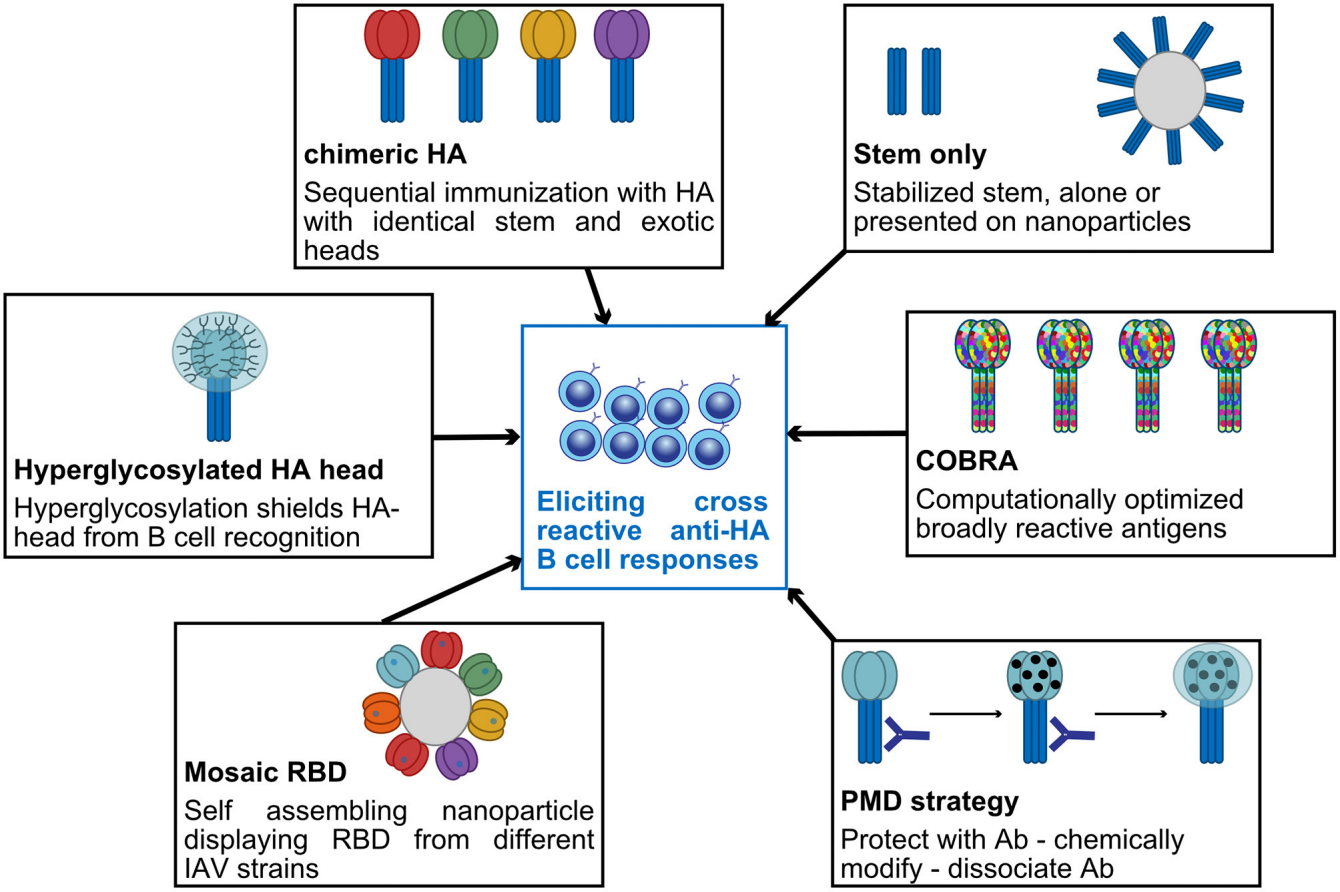
Summary of some promising strategies currently used to elicit broadly cross-reactive anti-HA B-cell responses.

HA is the most abundant glycoprotein on the influenza virion surface and is crucial for host viral entry by binding to the terminal sialic acid residues on epithelial cells, resulting in fusion of viral and host cell membranes. HA is a trimer consisting of a globular head, harboring the receptor binding site, and an elongated stem region (12). Even though stem-specific B cells and antibodies are generated during infection and vaccination, the HA head is the main target of neutralizing antibodies. However, possibly due to its immunodominance (13), the head is subjected to higher rate of evolution (2.2–4.4 times) than the stem (14, 15). Intriguingly, while in animals, at least 12 mutations are necessary to drive full escape from immune sera (16), in humans, it appears that the polyclonal response can be extremely focused on one antigenic site (17–19). For example, in a circulating span of 35 years in humans, a single amino acid substitution at only seven sites in HA head beside the receptor binding site (RBS) was enough to drive major antigenic change in H3N2 (17, 20). HA stem, as a target for universal influenza vaccine, has gained enormous traction in recent years. One could argue that the stem region is inaccessible to B cells and antibodies (21). However, a study using a broad neutralizing antibody showed that nearly 75% of the HA on pandemic H1N1 is bound by a stem-specific mAb (22). There is an urgent need to introduce universal vaccines, targeting conserved regions and providing lifelong protection. This review focuses on possible strategies for developing universal influenza vaccines, mainly based on HA. Such strategies are summarized in Figure 1.

## Hemagglutinin Stem—A Promising Universal Vaccine Target

HA stem has been an important candidate for development of universal vaccines because the stalk region is relatively conserved and evolves much slower and accommodate less amino acid substitutions as compared to the head domain. This could be due to minimal antibody pressure from low amount of circulating anti-stem antibodies (23, 24) and low tolerance to mutations in the stalk domain, which can lead to loss of viral fitness (25, 26), even though partial escape mutations in the stem can be generated (27). However, amino acid substitutions in the stalk have been reported to minimally affect the neutralization capacity of human cross-reactive, anti-stalk monoclonal antibodies (14, 28).

HA stem antibodies protect by (i) preventing viral entry by blocking the fusion of host cell membrane and viral membrane (29), (ii) reducing viral egress by blocking neuraminidase activity through steric hindrance (30–32), and (iii) FcR-mediated induction of antibody-dependent cellular cytotoxicity (ADCC), antibody-dependent cellular phagocytosis and reactive oxygen species production (33–35). Several human-derived broadly neutralizing anti-stem antibodies have been identified against either influenza group 1 (36–39) or group 2 (38, 40–42) or both groups (40, 43–50) or even against both influenza A and B subtypes (51). The identification of these antibodies was an incentive to develop vaccines, which are discussed below.

## Hemagglutinin Stem—Human Immune Responses

In humans, memory B cells (Bmem), and antibodies against HA stem are subdominant and present in low levels. Analysis of serum samples from 202 healthy individuals collected between 2004 and 2010 revealed that anti-stem antibodies of group 1 specificity is found in 84% of the population (52); however, their level, as measured in human Intravenous Immunoglobulin preparations, is very low (23).

Ellebedy and colleagues (53) found that immunization with H5N1, which is not currently circulating in humans, boosted cross-reactive antibody response toward HA stem, when compared to seasonal vaccines. Because of the existence of lower levels of H5 head-specific Bmem as compared to stem-specific Bmem, H5N1 vaccination led to recruitment of stem-specific Bmem, their expansion, and antibody production. On the contrary, boosting with the same HA favors anti-head responses (53). Another study found that nearly 6 out of 10 individuals have Bmem specific between group 1 and 2 HA (50). Indeed, it appears that baseline levels of H5–H1 cross-reactive Bmem and H1-specific Bmem are no more than 2-fold different (54). However, after priming with an H5 DNA plasmid vaccine and boosting with A/Indonesia/05/2005 monovalent-inactivated virus, both head and stem Bmem were expanded but only head-specific Bmem persisted, while stem-specific Bmem expanded and contracted rapidly (50, 54). Finally, Andrews et al. observed that immunization with group 1 virus (H5N1) elicited anti-stem memory responses exclusively against group 1, while group 2 (H7N9) induced high levels of cross-protective anti-stem memory B cell responses with diverse repertoire despite a lower overall response. This study in humans suggests the potential of group 2 based vaccines to provide a broader protection as compared to group 1 (55). While all these studies highlight the ability of individuals to generate stem-specific Bmem and plasmablasts, they all note a rapid contraction of stem-specific cells. This disconnect between cell numbers, longevity, and serum antibodies highlight the complexity of B-cell fate decision. Understanding how antigen specificity can influence cell differentiation is a crucial challenge for next generation vaccines.

## Sites Of Vulnerability In Hemagglutinin Head

Although HA stem is an excellent candidate for the development of universal vaccines, anti-HA stem titers in human correlates only with reduced viral shedding but do not predict the severity of influenza infection (56, 57). A recent study in humans indicated that a 2-fold increase in hemagglutination inhibition titer gave a 23.4% reduction in H1N1 infection risk, while the same increase in HA-stem-specific antibodies conferred only 14.2% reduction (58).

The globular head of the HA is target for most of the neutralizing antibodies, which prevents the viral entry by blocking the binding of RBS to sialic acid residues on host cell membrane (59). RBS is a shallow depression on HA head and consists of 130 loop, 150 loop, 190 helix, which are relatively conserved, and 220 loop, which is diverse among IAV subtypes (60, 61). Amino acid substitutions in the RBS determine host tropism, and specific substitutions are connected to altered receptor binding within subtype (62). Some RBS binding, broadly neutralizing antibodies have been identified, such as C05, S139/1, and F045-092, which neutralize within groups; CH65, 5J8, 2G1, and H5.3, which neutralize within subtype (63–71); and C12G6 and CR8033, which neutralize both influenza B lineages (51, 72).

Apart from RBS, broadly neutralizing antibodies have been identified against other conserved sites on HA head (73). An antibody (F005-126) which neutralizes 12 H3N2 subtypes by occupying the cleft formed by two HA head monomers and cross-linking them is known to prevent viral entry by blocking pH-induced HA conformational change (74). Bajic et al. found that subdominant antibodies can target an occluded epitope located on the lateral surface on HA head between two monomers using an H3 immunogen, hyperglycosylated on dominant epitopes. These antibodies protected against H3N2 challenge in an Fc-dependent manner (75). Similarly, two independent studies identified broadly neutralizing antibody, which bind hidden epitopes at HA trimer interface. These antibodies do not neutralize the virus but are suspected to disrupt the HA trimer integrity. Passive transfer experiments revealed that they protect mice from groups 1 and 2 viruses by preventing cell-to-cell viral spread or by FcγR or complement mediated effector mechanism (76, 77). HA exhibits “breathing” phenomenon at neutral or low pH where reversible separation of HA monomers exposes hidden epitopes to these specific antibodies (78–80). Vestigeal esterase domain is another possible HA target; it is located at the base of the HA head and can be target of broadly protective antibodies, which protect within subtypes (81, 82) and both lineages of influenza B virus (83). Like stem-directed antibodies, they protect through various mechanisms such as blocking viral egress, fusion, or by ADCC.

For most of the epitopes described above, we are just at the first step of the reverse vaccinology pipeline. However, there is hope that using some advanced *de novo* protein design tools, we will be able to create effective immune-focusing antigens (84, 85).

## Bioengineering Antigens To Refocus Immune Responses

### Headless Hemagglutinins

One of the obvious ways to increase the anti-stem antibody response is to remove HA head to circumvent its immunodominance (13, 86). Even though this sounds simple, removing the head and the transmembrane domain may cause conformational changes in the stem leading to loss of recognition by anti-stem antibodies (87). One of many successful attempts was achieved by creating stable trimeric mini-HA from H1N1, which retained similar binding affinity to two broadly neutralizing antibodies, CR9114 and CR6261, as that of full-length HA. When used to immunize non-human primates, the elicited antibodies competed with CR9114, induced ADCC, and neutralized H5N1 virus (88). Another study used H1HA10-Foldon and H3HA10-Foldon mini-HA, which generated neutralizing antibodies cross-reactive to both groups 1 and 2 IAV *in vitro* but with limited cross-group protection *in vivo* (89, 90). In contrast to these mini-HAs, an H5-based mini-HA produced and purified from *Escherichia coli* elicited anti-stem antibody responses and protection against both IAV subtypes (91). Pre-existing immunity plays a role in subsequent immune response to viral infection and vaccination (92). When tested in non-human primates who have been exposed to flu infection, mini-HA were shown to induce higher cross-protective antibody response as compared to seasonal trivalent inactivated vaccine, indicating their potential as a universal vaccine (93).

Ferritin is an iron storage protein which self-assembles into a nanoparticle consisting of 24 repetitive polypeptides, which can induce strong immune response and antigen presentation (94). Based on this, Yassine et al. engineered a nanoparticle containing intact HA stem from H1 (H1-ss-np), which generated anti-stem response almost 2-fold higher than that of trivalent inactivated vaccine. When immunized with H1-ss-np, mice and ferrets elicited broad antibody response against group 1 IAV as well as group 2 IAV, demonstrating heterosubtypic protection (95). Based on this, influenza H1 stabilized stem ferritin vaccine (H1ssF_3928) has entered a phase I ongoing clinical trial to evaluate its tolerability and immunogenicity in healthy adults. However, a thermostable and immunogenic nanoparticle vaccine containing group 2 H3 and H7 stem conferred only protection against homosubtypic viral challenge. Given the fact that these stem immunogens activates IgM BCR of unmutated common ancestor of the cross-reactive human anti-stem antibodies, the authors speculate that the lack of cross-group protection *in vivo* might be due to difference in BCR repertoire in mice and human (96).

### Chimeric Hemagglutinin

Chimeric HAs (cHA) are full-length HA with stem derived from human viruses and globular head from distinct, exotic HAs. Based on this concept, repeated immunization with cHA with head from different flu subtype and same H1 HA stem induced high titers of stem-reactive antibodies against homologous and heterologous viruses (97). Several such cHA constructs, which confer protection by eliciting stem Abs, have been developed for group 1, group 2, and Influenza B viruses, with some inducing long-lasting antibodies (98–105). An interim report on a clinical trial using a cHA prime boost strategy was recently released (106). In this study, healthy volunteers, with measurable baseline H1-stalk antibody levels, were boosted with cHAs. The sharpest stem-antibody level increase was obtained when challenging with cH8/1N1 in AS03 adjuvant intramuscularly. However, further immunization with other cHA did not additionally boost stem responses. To build upon these promising findings, more studies are needed to assess the longevity of these responses and their stability upon natural infection or seasonal immunization.

### Immune-Focusing Strategies

N-linked glycosylation on HA has been known to stabilize HA and shield virus from host immune response (107). Creating monoglycosylated HA or unmasking HA-stem of N-glycans could induce cross-strain neutralizing anti-stem antibodies (108, 109). Conversely, hyperglycosylating the HA head can also refocus response on stem (110). A recent study used “protect, modify, and deprotect” method to focus antibody response toward a stem epitope. In this strategy, after masking target epitope on stem with a monoclonal antibody, the surrounding undesired epitopes are chemically modified to reduce their antigenicity (111). On the other hand, other residues, outside stem, can also influence the neutralizing sensitivity to anti-stem antibodies by affecting HA-stalk stability and antibody access to stem epitopes (112).

### Vaccine Engineering for Cross-Protection

Kanekiyo et al. used a ferritin nanoparticle displaying a repetitive array of HA hyper variable receptor binding domains (RBD from different H1N1 strains). Using this, they could subvert B cell response from strain-specific immunodominant epitopes to conserved, shared epitopes. Since cross-reactive B cells can recognize conserved epitope from an heterogenous array of RBD, these cells get preferentially activated due to avidity advantage and could selectively activate pre-existing cross-reactive Bmem. One of the antibodies generated after immunization, 441D6, binds to a conserved epitope, which lies opposite to RBS and protects against H1N1 strains spanning from 1977 to 2009 (113, 114). Anderson et al. generated cross-reactive antibody responses by injecting a mix of DNA vaccines containing HA genes from six members of group 1 IAV, which was further enhanced by inclusion of an antigen presenting cell targeting unit specific for MHCII, thus favoring BCR cross-linking (115). Another strategy to elicit broadly reactive antibodies within IAV subtypes is to use computationally optimized broadly reactive antigens (COBRA), which are displayed on virus like particles or expressed in live attenuated influenza vaccine. This approach uses multiple rounds of consensus generation to aggregate HA epitopes of IAV from different time periods. Such vaccines elicit polyclonal B cell responses and was shown to protect within subtypes (116–123). Combining several COBRA vaccines could confer heterosubtypic protection.

### M2e-Based Vaccines

M2 is an ion channel with its ectodomain (M2e) exposed on virion and infected cell surfaces (124). M2e is quite conserved across IAV; therefore, it has historically been considered as an ideal universal vaccine candidate (125). The mechanism of M2e-mediated protection is debated with both antibodies and T cells being important players (126–128). Several approaches have been undertaken to increase M2e immunogenicity, using VLP or different adjuvants (129–131). Of note, it appears that M2e antibodies act via effector functions and thus are infection permissive, making M2e vaccines more suitable when used in combination with others.

## Neuraminidase—The Emerging Player

IAV NA as vaccine target has been neglected for decades, despite early discovery of potent anti-viral activity of NA antibodies (132). Even more surprisingly, NA amount in licensed vaccines varies enormously and is not checked by manufactures or regulatory authorities (133). Exciting new studies strongly point to a major role for anti-NA antibodies in protecting from disease and as the best correlates of protection (56, 134, 135). Critically, Chen et al. identified a number of human NA antibodies that cross-protected mice in therapeutic and prophylactic setting (136). Even more promising, several broadly neutralizing anti-NA mAbs have been isolated from an infected patient. These mAbs, directed to NA active site, demonstrated an unusual breadth in binding several IAV and IBV NA and mediating cross-neutralization and cross-protection *in vivo* (137). Still, despite some early studies, we do not know enough about NA antigenicity and the immunodominance of its antigenic sites (138–141). By applying some of the methods that allowed us to study in detail anti-HA responses, we should be able to break down anti-NA responses and identify promising universal vaccine candidates.

## Conclusions—Know What We Do Not Know

Bioengineering and design of epitope-focused immunogens is proceeding at an incredible speed in influenza and other fields. Several promising immunogens are now in clinical trials and, hopefully, will be available to the public soon, as long-lasting universal vaccines. It is, however, crucial to understand more about the basics of B cell responses to interpret results and inform on vaccination policies.

Introduction of pandemic H1N1 2009 virus showed that most individuals, with low serological anti-stem antibodies, were able to mount a stem-directed response, but repeated vaccinations skewed the immune response back to the immunodominant head (21). It will be critical to understand when, in which order and how often give universal vaccines to appropriately boost stem response. Andrews et al. demonstrated that novel B cells specific for variable epitopes have a different phenotype compared to reactivated Bmem specific for stem (142). To maximize success, efforts will need to be put in understanding how B-cell specificity can influence their programming and differentiation. Furthermore, it is still unclear how much of stem-specific antibodies are required for optimal protection from a drifted or heterologous virus. Table 1 summarizes what we know about antibody responses to the major targets on IAV and how seasonal vaccination is able to boost those responses.

**Table 1:** Characteristics of antibody responses to current universal vaccine targets and ability of seasonal vaccination to recall memory B cells and specific antibodies.

	**Antibodies**			**Seasonal vaccination**	
	**Broadly cross reactive**	**Neutralizing**	**Act via effector functions**	**Recall memory B cells**	**Elicit antibodies**
HA head	–	+	– (33)	+	+
HA head conserved targets	+	+/–^a^	+/–^b^	?^c^ (73, 143)	?^d^ (143)
HA stem	+	+	+ (33)	–/+^e^ (21, 144)	–/+^e^ (21)
NA	+	+/–^f^ (141)	+ (145)	–^g^ (136)	+/–^g^ (146, 147)
M2e	+	– (148)	+ (149)	–	–

a *HA head conserved targets comprise lot of different targets. Neutralization ability depends on the target*.

b *See in the body of this review for references, depending on the target*.

c *Not many studies address this question. It appears that vaccination with newly introduced viruses might recall these B cells*.

d *Not many studies are addressing this issue, which is probably dependent on the target*.

e *Stem-specific memory B cells are mainly recalled and antibodies induced when new viruses are introduced (for example with H1N1/pdm2009)*.

f *NA antibodies usually have NA-inhibition activity, which correlates well with plaque reduction but are not neutralizing, by definition*.

g *NA-B cells and antibodies are most likely not properly boosted, after seasonal vaccination, due to poor vaccine formulation, with variable/low NA amount*.

Finally, but not less important, new vaccines platforms are constantly being tested. RNA-based vaccines have shown exciting results when expressing influenza proteins, at least in animals [reviewed in (150)]. Some of the engineered vaccines discussed in this review could be delivered as RNA vaccines, alone or in combination, possibly avoiding clearance from pre-existing antibodies. Other novel, slow-release, vaccine formulations could help refocusing immune responses to subdominant targets (151–153).

We are just entering an exciting season of clinical trials, and while expectations are really high, we should not be discouraged if some of the early attempts fail but rather persevere in the quest for a universal and long-lasting vaccine.

## Author Contributions

NM and DA researched the literature and wrote the manuscript.

### Conflict of Interest

The authors declare that the research was conducted in the absence of any commercial or financial relationships that could be construed as a potential conflict of interest.
